# Therapeutic Exercise and Pain Neurophysiology Education in Female Patients with Fibromyalgia Syndrome: A Feasibility Study

**DOI:** 10.3390/jcm9113564

**Published:** 2020-11-05

**Authors:** Luis Ceballos-Laita, María Teresa Mingo-Gómez, Francisco Navas-Cámara, Elena Estébanez-de-Miguel, Santos Caudevilla-Polo, Zoraida Verde-Rello, Ana Fernández-Araque, Sandra Jiménez-del-Barrio

**Affiliations:** 1 Department of Surgery, Ophthalmology and Physiotherapy, Faculty of Health Sciences, University of Valladolid, 42004 Soria, Spain; luis.ceballos@uva.es (L.C.-L.); tmingo@cir.uva.es (M.T.M.-G.); fjnavas@bio.uva.es (F.N.-C.); zoraida.verde@uva.es (Z.V.-R.); afa@enf.uva.es (A.F.-A.); 2 Department of Physiatrist and Nursey, Faculty of Health Sciences, University of Zaragoza, 50010 Zaragoza, Spain; elesteba@unizar.es (E.E.-d.-M.); scp@unizar.es (S.C.-P.)

**Keywords:** fibromyalgia, chronic pain, exercise, physical therapy modalities, patient education

## Abstract

Background: We compared the effects of therapeutic exercise (TE) combined with pain neurophysiology education (PNE) to those of TE in isolation on pain intensity, general fibromyalgia impact, mechanical pain sensitivity, pain catastrophizing, psychological distress and quality of life in women with fibromyalgia syndrome (FMS). Methods: A feasibility study with a 3 month follow-up was designed. Thirty-two patients with FMS were randomly assigned to PNE + TE group (*n* = 16) or to TE group (*n* = 16). Both groups received 30 sessions of TE (3 per week), and the PNE + TE group received eight face-to-face educational sessions. The measuring instruments used were the visual analogue scale, a standard pressure algometer, the Revised Fibromyalgia Impact Questionnaire, the Pain Catastrophizing Scale, the Hospital Anxiety and Depression Scale and the Health Assessment Questionnaire. Results: The PNE + TE group showed a statistically significant decrease on pain intensity compared to TE group at short term (*p* = 0.015). No between-groups differences were found for mechanical pain sensitivity, general fibromyalgia impact, pain catastrophizing, psychological distress or quality of life (*p* > 0.05). Conclusions: The combination of PNE and TE was more effective than TE for reducing pain intensity in the short-term. No differences were found for psychological distress, pain catastrophizing and quality of life after the intervention or at 3 months of follow-up.

## 1. Introduction

Fibromyalgia syndrome (FMS) is a rheumatological disorder characterized by widespread pain and tenderness. Pain is the main symptom in FMS [1,2,3] and is commonly associated with psychological distress such as anxiety and depression, affecting quality of life [4]. Several studies investigated the prevalence of FMS in different countries. The global prevalence of FMS has been established in the general population as between 0.2 and 6.6% and has been shown to be higher among women [5].

Pharmacological treatments are widely used for FMS. Studies based on pharmacological interventions reported some adverse events, such as drug intolerance, secondary effects and several complications from continuous intake [6]. In addition, the effects of these treatments disappear as soon as the intervention finishes [7]. Currently, clinical guidelines are focused on non-pharmacological treatments and recommend a multidisciplinary approach for FMS [7,8]. The latest European League Against Rheumatism (EULAR) guidelines recommend graded therapeutic exercise (TE) combined with patient education for FMS management [7].

Exercise has shown positive effects in patients with FMS [9,10,11,12,13,14,15,16,17]. Previous studies have demonstrated that strengthening and aerobic exercises in isolation were effective in reducing FMS symptoms [9,11,15,17]. The comparison of both types of exercise showed similar effects on reducing pain intensity and improving quality of life [10,11,15]. In this sense, the combination of aerobic, strengthening and stretching exercises achieved better results than aerobic or strengthening exercises in isolation on symptoms and quality of life [16,18].

Pain neurophysiology education (PNE) is an educational intervention for patients with chronic pain. PNE uses neurophysiological information to teach patients that pain can be overprotective and completely real, even in the absence of tissue injury. The goal of PNE is to change patients’ beliefs, considered as a preliminary step to changing behavior [19,20,21]. PNE combined with TE has shown to be effective in reducing pain and increasing physical function in patients with chronic low back pain [22,23] but has never been taken into consideration in patients with FMS.

Although clinical guidelines recommend the combination of patient education and graded physical exercises, there is a lack of high-quality studies evaluating the effects of PNE combined with TE. Thus, the present study aimed to analyze the effects of TE combined with PNE compared to those of TE on pain intensity, mechanical pain sensitivity, catastrophizing thoughts and feelings, psychological distress and quality of life in women with FMS.

## 2. Material and Methods

### 2.1. Study Design

A feasibility study conceptualized as a non-inferiority design with two intervention groups (PNE+TE group and TE group) was conducted with repeated measures. The study was carried out at the University of Valladolid, Faculty of Health Sciences. This study was developed according to Consolidated Standards of Reporting Trials (CONSORT) guidelines. All procedures were approved by the Clinical Research Ethics Committee of Burgos-Soria (CEIC 1903) and registered at clinicaltrials.org (NCT03641495). The selection and recruitment were conducted following the declaration of Helsinki and all patients provided written informed consent prior to enrolment.

### 2.2. Participants

All participants (*n* = 36) were female patients diagnosed with FMS referred from medical doctors and from the fibromyalgia association FIBROAS (Soria, Spain) between January 2018 to September 2019.

Inclusion criteria were: (1) woman diagnosed with FMS by a rheumatologist according to the latest American College of Rheumatology (ACR) classification criteria [24]; (2) aged from 20 to 65 years; (3) and agreement to attend therapy sessions. Exclusion criteria were: (1) presence of cardiovascular, respiratory, metabolic, neurological, rheumatic, renal or hepatic diseases that could limit exercise; (2) severe somatic diseases (e.g., cancer); (3) psychiatric diseases (e.g., psychotic); (4) pregnancy or lactation; (5) changes in the pharmacologic therapy last three months or during the period of the study; (6) exercise or previous physiotherapy treatments within the last three months; (7) exercise contraindications; or (8) inability to understand the questionnaires.

### 2.3. Sample Size

The sample size calculation was based on pain intensity as a primary outcome using Minitab^®^ 13.0 program. For the differences in mean values between groups, 2 cm was the minimum relevant change considered in the visual analogue scale (VAS). The standard deviation was based on a previous study in patients with FMS. Assuming a standard deviation of 1.8 and a between-mean difference of 2 cm, and estimating a two-tail test a level of significance of 0.05, a power of 0.8 and a follow-up loss rate of 15%, 16 participants were required for each group.

### 2.4. Randomization

Participants who fulfilled the eligibility criteria were randomly assigned to PNE + TE group or to TE group. Concealed allocation (ration 1:1) was performed by an external examiner using the Research Randomizer (Version 4.0) computer software (www.random.org).

### 2.5. Interventions

Both groups received the TE intervention by a physiotherapist with more than 5 years of experience with FMS patients and chronic pain. All participants received a total of 30 sessions (3 sessions per week for 10 weeks). The PNE+TE group also received 8 sessions (once a week for 8 weeks) of an educational program based on PNE by a medical doctor expert in pain neurophysiology. The treatment adherence was registered with a list of attendance in each intervention group.

### 2.6. Therapeutic Exercise

Both groups received 3 TE sessions per week for 10 weeks. The sessions were carried out by a physiotherapist blinded to the group allocation. The TE program included an active warm-up including low intensity movements and dynamic stretching, a central part with aerobic training and strengthening exercises of the major muscles and a cool-down part including static stretching and respiratory exercises. Each session lasted 60 min.

The active warm-up was performed at a light level of intensity, prior to the central part. The warm-up protocol was based on joint mobility exercises, active stretching and submaximal repetitions of the strengthening exercises designed for each session.

In the central part, the aerobic exercises (cycling, walking and playing games) were adapted to a gradual progression from low intensity to moderate intensity, equivalent to 40–60% of age adjusted maximum heart rate (220–age) [25]. To achieve the gradual progression, the cardiorespiratory fitness of each participant was calculated based on the maximum heart rate and was monitored during the aerobic exercise. The strengthening exercises were performed for the major muscle groups (gluteus, quadriceps femoris, hamstrings, biceps brachii, triceps brachii, deltoid and latissimus dorsi) according to the American College of Sports Medicine (ACSM) guidelines [25]. Major muscles were trained in circuits of 4 different stations, with 3 sets of 10 to 12 repetitions that were chosen for each session. The strengthening exercises were tailored to functional activities, such as sitting, squatting, climbing and lifting, and different progressions were made by adding soft elastic bands and dumbbells to individually adjust the intensity of each exercise. The intensity of the strengthening exercises was calculated concerning the assessment process, and it was 50% of the one-repetition maximum (1 RM) calculated by submaximal testing at the beginning. Patients were asked to progressively increase by 10% the intensity of the strengthening protocol according to the individual tolerance. The intensity was increased when patients were comfortable with the exercises for 2 weeks. The intensity of the exercises was reduced in cases of post-exertion pain or fatigue [26,27].

The cool-down part consisted of 3 sets of 30 s of static stretching of the major muscles trained and breathing techniques. The exercise protocol used is shown in Appendix A.

The type of exercises and the intensity selected for each type of exercise were developed according to the findings described by Busch et al. [27]. All the exercises were patient-tailored and controlled by a physiotherapist to ensure the patient comfort and safety and to minimize possible adverse events.

### 2.7. Pain Neurophysiology Education

The PNE+TE group received 8 face-to-face educational sessions. The sessions were carried out by a medical doctor. The PNE program was performed based on the recommendations described by Butler and Moseley [28]. The sessions explained the physiology of the nervous system in general and the pain system in particular. Each session lasted from 30 to 45 min.

The topics explained during the education sessions included the characteristics of acute versus chronic pain, the purpose of acute pain, how acute pain originates in the nervous system, how pain becomes chronic and potential sustaining factors of central sensitization, such as emotions, stress, illness perceptions, pain cognitions and pain behavior. Acute nociceptive mechanisms were explained first and then were contrasted with central sensitization processes. Different examples and metaphors were used to clarify the contents. The education was presented verbally and visually. All the questions or doubts of the participants were answered during the educational sessions or at any moment during the rest of the study. The patients were encouraged to apply the new knowledge in daily life.

### 2.8. Outcome Measures

**Figure 1:**
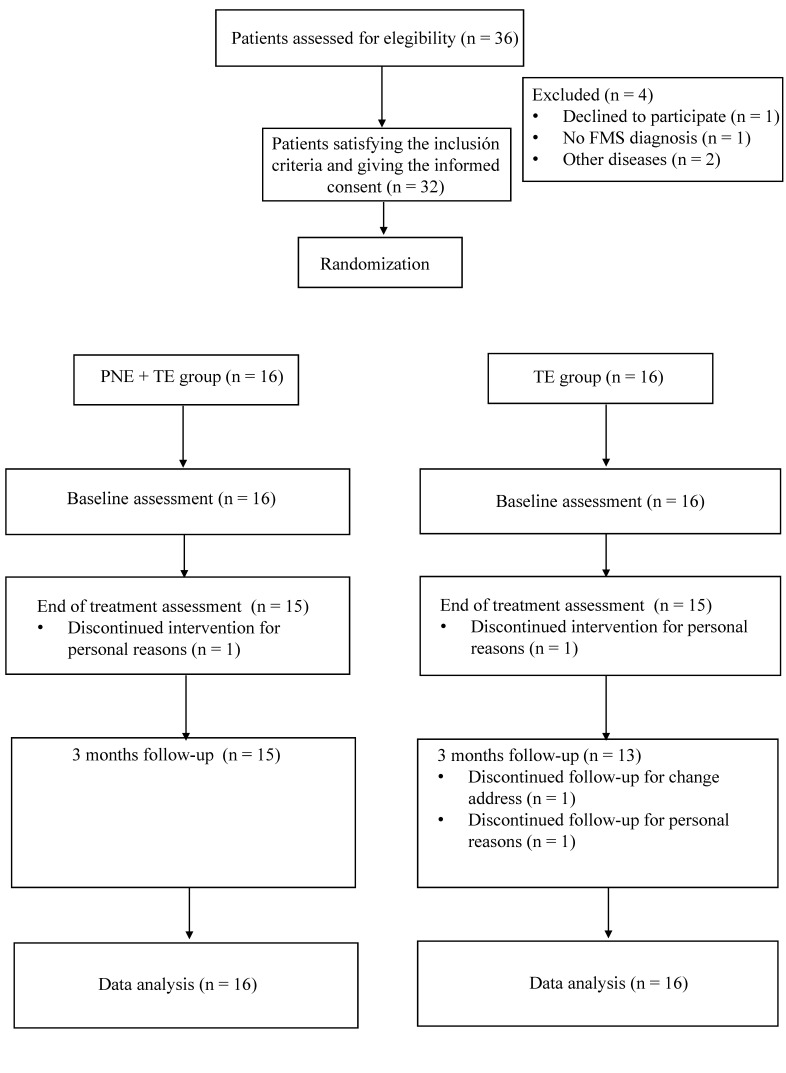
Diagram clinical trial process.

Sociodemographic information, including gender, age, height, weight, body mass index (BMI) and years since diagnosis were registered for descriptive purposes. The primary outcome variables were pain intensity and mechanical pain sensitivity. Secondary outcome variables were general fibromyalgia impact, psychological distress, pain catastrophizing and quality of life. All the outcome variables were measured at baseline (T0), one week after the 10 week intervention (T1) and at the 3 month follow-up after T1 (T2). T1 was performed one week after finishing the intervention to avoid any related fatigue. Figure 1 shows the clinical trial process diagram. The evaluators were three physiotherapists blinded to treatment allocation.

Generalized pain intensity was recorded using a 10 cm visual analogue scale (VAS). The ends represented the extreme expressions in which 0 represented “no pain” and 10 “the most intense pain imaginable.” Patients were asked to report pain intensity in the last 3 days. The VAS has been widely used for assessing pain intensity in patients with FMS. This scale has shown an intraclass correlation coefficient (ICC) of 0.97 for patients with chronic pain [29]. The Minimal Clinically Important Change (MCID) of VAS has been stated as 1.5–2 cm for chronic pain [30].

Mechanical pain sensitivity was assessed using a standard pressure algometer (Psymtec, FPK 20). The 18 tender points registered in the 1990 ACR criteria were measured [31]. The tender points were: right and left occiput (P1 and P2), low cervical (P3–P4), trapezius (P5–P6), supraspinatus (P7–P8), second rib (P9–P10), lateral epicondyle (P11–P12), gluteal (P13–P14), greater trochanter (P15–P16) and knee (P17–P18). The pressure pain threshold of each tender point was determined by applying increasing pressure with the algometer. The pain threshold is defined as the minimal amount of pressure at which the sense of pressure first changes to pain [32]. Patients were asked to stop the test when the pressure became a clear sensation of pain. The number of tender points and the algometer score were analyzed after the measurements. A point was considered tender if pain from pressure required ≤4 kg/cm^2^. The algometer score was calculated as the sum of the pain-pressure values obtained for each tender point [33].

The general fibromyalgia impact was assessed by a Spanish version of the Revised Fibromyalgia Impact Questionnaire (FIQ-R). The FIQ-R assesses function, impact and symptoms related to FMS. The FIQ-R ranges from 0 to 100, with 100 indicating maximum FMS impact. This questionnaire has been shown to be reliable with an ICC superior to 0.7 [34].

Pain catastrophizing was evaluated using the Spanish version of the Pain Catastrophizing Scale (PCS). This scale consists of 3 subscales assessing rumination, magnification and helplessness. Each item rated from 0 (no negative thoughts or feelings) to 3 (maximum negative thoughts or feelings). This scale has shown an ICC of 0.84 [35].

Psychological distress was evaluated using the Spanish version of the Hospital Anxiety and Depression Scale (HADS) [36]. This scale consists of 2 subscales measuring depression and anxiety. HADS is a validated questionnaire that consists of 14 items, each rated from 0 (no distress) to 3 (maximum distress). The cut-off score for the presence of anxiety and depressive symptoms is ≥8. The sensitivity and specificity for this cut-off are both 0.80 [37].

The quality of life was measured using the Spanish version of the Health Assessment Questionnaire (HAQ9). The scale assesses the difficulty in performing daily living activities (dressing, rising, eating, walking, hygiene, reach, grip and usual activities). The HAQ consists of 20 items, each rated from 0 (no disability) to 3 (completely disabled). This scale has shown an ICC ranging from 0.87 to 0.99 [38].

### 2.9. Statistical Analysis

SPSS software version 20.0 for Windows was used for statistical analysis (SPSS Inc, Chicago, USA). The statistical analysis was conducted according to intention-to-treat (ITT). Mean and standard deviations were calculated for quantitative variables. Normal distribution of the variables was analyzed using the Shapiro–Wilk test (*p* > 0.05). Baseline demographic and clinical variables were compared between groups using a Student’s t test or the Mann–Whitney U test according to the normally distributed data or non-normally distributed data.

The group by time effects between both groups (PNE + TE and TE) and time points (baseline, end of treatment, and 3 months of follow-up) were calculated using two-way repeated measures analysis of variance ANOVA. A *p*-value <0.05 was considered statistically significant. The effect size (Cohen’s *d*) was also calculated to estimate the magnitudes of the within-group differences. The magnitude of the difference was classified as small if the value of Cohen’s d ranged from 0.2 to 0.5; as moderate if it ranged from 0.5 to 0.8; and as large if Cohen’s d was greater than 0.8. Moderate and large magnitudes of effect size were considered indicators of appropriate statistical power [39].

## 3. Results

**Figure 2:**
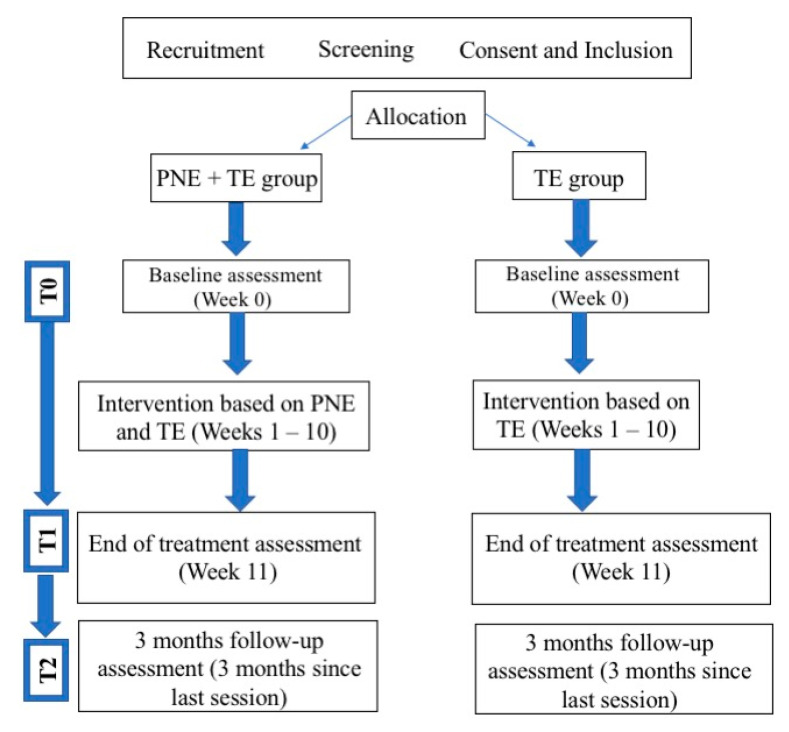
Flowchart diagram.

In total, 36 women were recruited, and 32 women (52.6 ± 10.4 years) diagnosed with FMS who met the inclusion criteria were enrolled in the clinical trial. They were randomly assigned to the PNE + TE group (*n* = 16) or the TE group (*n* = 16). Four patients dropped out during the study for personal reasons. A flowchart of the recruitment and follow-up of participants is shown in Figure 2. The M ± SD for demographic and clinical characteristics and differences between groups at T0 are shown in Table 1. There were no statistically significant differences between groups in any of the sociodemographic or dependent variables at baseline (*p* > 0.05).

After the intervention, the ANOVA analysis showed a significant group by time interaction for pain intensity (F = 6.64; *p* = 0.015) at T1. The PNE + TE group showed a greater decrease of pain intensity than the TE group (Δ −2.16; 95% CI: −3.87 to −0.45). The between-groups difference was not maintained at T2 (F = 2.39; *p* = 0.132). No statistically significant between-group differences were found for the number of tender points (T1: F = 0.01; *p* = 0.898; T2: F = 0.896; *p* = 0.351), the algometer score (T1: F = 0.11; *p* = 0.271; T2: F = 2.65; *p* = 0.114), FIQ-R (T1: F = 0.03; *p* = 0.855; T2: F = 3.53; *p* = 0.07), PCS (T1: F = 0.02; *p* = 0.884; T2: F = 0.28; *p* = 0.600), HADS (T1: F = 0.21; *p* = 0.649; T2: F = 0.30; *p* = 0.588) or HAQ (T1: F = 2.26; *p* = 0.143; T2: F = 1.49; *p* = 0.231). Table 2 provides before, after and follow-up data and between-groups differences for all the dependent variables.

The within-group change scores and within-group effect sizes for all the variables measured are shown in the Supplementary Data.

## 4. Discussion

To the best of our knowledge, this was the first study to investigate the effects of PNE combined with TE compared to TE in women with FMS. The results of our study showed that the PNE + TE group showed a greater improvement in pain intensity after the intervention compared to TE group. No between-groups differences were found for the number of tender points, mechanical pain sensitivity, impact of FM, pain catastrophizing, psychological distress or quality of life. Both groups reported improvements in the number of tender points, mechanical pain sensitivity and general fibromyalgia impact.

Greater improvements were achieved for pain intensity at T1 and at T2 in the PNE + TE group. The change achieved for pain intensity in the PNE + TE group was higher than the MCID stated for patients with chronic pain [30]. The number of tender points and the mechanical pain sensitivity decreased after both interventions. These results are in agreement with previous studies that showed that PNE or its combination with exercise reduce pain and mechanical pain sensitivity in patients with chronic pain [20,40,41,42].

It has been described that cognitions and emotions can modulate pain through descending pathways because they are directly connected to different brain areas, such as the limbic system [43]. Patients diagnosed with FMS that do not understand the mechanism of chronic pain usually present negative emotions that could facilitate pain through the descending pathways [44]. PNE has been shown to change pain cognition and normalize emotions and beliefs about pain [20], improving endogenous nociceptive inhibition in patients with FMS [41]. In this way, the inclusion of education about chronic pain in our study could have produced positive effects on central pain processing, explaining the greater decrease in pain intensity achieved by PNE + TE group.

General fibromyalgia impact, pain catastrophizing, psychological distress and quality of life variables fluctuated somewhat over time, but no statistically significant changes between groups were found. The results of this study showed that PNE + TE is not more effective than TE for improving these variables. According to this, PNE may not contribute to greater benefits in these variables. These results achieved are in accordance with recent publications which concluded that education in isolation seems not to produce improvements in general fibromyalgia impact, pain catastrophizing and quality of life [42,45].

Although education is a promising approach that allows the therapist to interact and to explain certain information with different examples, the patients’ appreciation and understanding of the information should be assessed. The results reported in this study could be related to the nonspecific factors associated with the face-to-face sessions that were not measured, such as the patient’s emotional processing of the encounter with the healthcare professional, the quality of the therapeutic alliance, the empathic understanding from the clinician and treatment preferences [46,47].

From a clinical perspective, the results achieved in this study showed that the combination of patient education through PNE plus exercise therapy seems to produce better benefits in the short-term for pain intensity than exercise in isolation. These results are in accordance with the latest EULAR guidelines for the management of FMS [7]. PNE seems to be an effective patient education intervention and its combination with TE could improve pain intensity. Patients need a thorough understanding of the course of symptoms to decide on the therapeutic approach. The results of this study may help in the decision-making process. In this way, patients with widespread pain treated with exercise combined with education could decrease pharmacological interventions in the short- and medium-terms.

Several limitations need to be considered. First, the small sample size included in the study, which makes difficult the extrapolation of the results. A larger sample could be more likely to determine differences between the two interventions in the variables that showed no statistical changes. Secondly, all patients were recruited from a fibromyalgia association and showed the motivation to participate in the study; other selection mechanisms should be applied. Third, there was a lack of a control group to control the changes when no intervention was applied; a lack of healthy control group to understand the effects of this program in healthy subjects; and a lack of PNE group who performed PNE in isolation to report the effects of the specific PNE intervention. Fourth, only women were included in the study, so results cannot be extrapolated to the male population. Fifth, a 10 week intervention could be not enough for the management of a chronic condition. Finally, only short-term and medium-term effects were assessed. Future studies should investigate the long-term effects of PNE combined with TE in the male population, and its inclusion in a multimodal approach.

## 5. Conclusions

The results of this study showed that the combination of PNE and TE was more effective than TE for reducing pain intensity at short-term. No differences were found for psychological distress, pain catastrophizing and quality of life after the intervention or at 3 months of follow-up.

## Figures and Tables

**Table 1:** Baseline and sociodemographic data.

	PNE + TE Group (*n* = 16)	TE Group (*n* = 16)	*p*-Value
Age (years)	52.13 ± 10.31	53.00 ± 10.68	0.818
Height (cm)	159.66 ± 5.08	159.35 ± 5.87	0.874
Weight (Kg)	76.56 ± 16.14	66.34 ± 12.34	0.052
BMI (Kg/cm^2^)	30.10 ± 6.69	26.17 ± 4.99	0.070
Years since diagnosis	12.37 ± 8.64	11.12 ± 8.10	0.676
VAS last three days (cm)	6.91 ± 1.62	7.11 ± 1.65	0.732
Number tender points	17.43 ± 1.63	17.37 ± 1.02	0.898
Algometry (Kg/cm^2^)	33.78 ± 12.70	33.57 ± 9.88	0.959
FIQ-R	60.98 ± 18.03	60.26 ± 15.05	0.982
PCS	24.75 ± 12.31	22.81 ± 8.51	0.609
HADS	18.06 ± 7.87	18.50 ± 7.17	0.871
HAQ	9.06 ± 4.65	9.88 ± 3.57	0.584

ITT: intention-to-treat; PNE+ET: pain neurophysiology education + exercise therapy; ET: exercise therapy; BMI: body mass index; VAS: visual analogue scale; FIQ-R: Revised Fibromyalgia Impact Questionnaire; PCS: Pain Catastrophizing Scale; HADS: Hospital Anxiety and Depression Scale; HAQ: Health Assessment Questionnaire.

**Table 2:** Descriptive data, between group statistical significance and score changes in ITT analysis.

	Baseline (T0)	End of Treatment (T1)	Between-Group Score Changes	Between-Group *p*-Value	3-Months Follow-Up (T2)	Between-Group Score Changes	Between-Group *p*-Value
VAS last three days (0–10 cm)							
PNE + TE group	6.91 ± 1.62	3.99 ± 2.22	−2.16 (−3.87, −0.45)	F = 6.64	4.21 ± 2.87	−1.39 (−3.23, 0.44)	F = 2.39
TE group	7.11 ± 1.65	6.15 ± 2.50		*p* = 0.015	5.61 ± 2.16		*p* = 0.132
Number of tender points							
PNE + TE group	17.43 ± 1.63	14.5 ± 4.11	0.63 (−0.92, 1.04)	F = 0.01	13.68 ± 4.12	−1.25 (−3.94, 1.44)	F = 0.896
TE group	17.37 ± 1.02	15.56 ± 3.68		*p* = 0.898	14.93 ± 3.29		*p* = 0.351
Algometer score (Kg/cm^2^)							
PNE + TE group	33.78 ±12.70	53.60 ± 19.10	7.56 (−6.21, 21.35)	F = 0.11	58.14 ± 22.38	11.35 (−2.88, 25.59)	F = 2.65
TE group	33.57 ± 9.88	46.03 ± 19.08		*p* = 0.271	46.78 ± 16.83		*p* = 0.114
FIQ-R PNE + TE group TE group	60.98 ± 18.03 60.26 ± 15.05	51.88 ± 21.91 52.23 ± 21.69	−0.35(−16.09,15.39)	F = 0.03 *p* = 0.855	37.66 ± 20.89 48.56 ± 21.25	−10.9 (−26.12,4.31)	F = 3.53 *p* = 0.07
PCS Total score							
PNE + TE group	24.75 ± 12.31	18.69 ± 11.49	−0.56 (−8.35, 7.23)	F = 0.02	17.75 ± 11.86	−2.00 (−9.70, 5.70)	F = 0.28
TE group	22.81 ± 8.51	19.25 ± 10.03		*p* = 0.884	19.75 ± 9.32		*p* = 0.600
PCS Rumiation							
PNE + TE group	8.62 ± 3.93	6.63 ± 4.17	−0.5 (−3.36, 2.36)	F = 0.12	6.31 ± 4.06	−1.00 (−5.70, 9.70)	F = 0.53
TE group	8.69 ± 3.36	7.13 ± 3.75		*p* = 0.724	7.31 ± 3.66		*p* = 0.470
PCS Magnification							
PNE + TE group	5.06 ± 3.02	3.50 ± 2.85	−0.31 (−2.31, 1.68)	F = 0.10	3.50 ± 3.07	−0.31 (−2.27, 1.65)	F = 0.10
TE group	4.13 ± 2.39	3.81 ± 2.88		*p* = 0.752	3.81 ± 2.31		*p* = 0.748
PCS Helplessness							
PNE + TE group	11.06 ± 5.96	8.56 ± 5.18	0.31 (−3.26, 3.89)	F = 0.03	7,94 ± 5.38	−1.00 (−4.61, 2.61)	F = 0.32
TE group	10.00 ± 4.42	8.25 ± 4.72		*p* = 0.860	8.94 ± 4.58		*p* = 0.576
HADS total score							
PNE + TE group	18.06 ± 7.87	14.56 ± 9.18	−1.43 (−7.83, 4.95)	F = 0.21	14.19 ± 7.95	1.31 (−3.58, 6.21)	F = 0.30
TE group	18.50 ± 7.17	16.00 ± 8.50		*p* = 0.649	12.87 ± 5.36		*p* = 0.588
HADS anxiety							
PNE + TE group	9.75 ± 3.56	8.44 ± 4.41	−0.43 (−3.54, 2.67)	F = 0.08	7.56 ± 3.66	0.37 (−2.07, 2.82)	F = 0.09
TE group	10.31 ± 3.45	8.88 ± 4.19		*p* = 0.776	7.19 ± 3.08		*p* = 0.756
HADS depression							
PNE + TE group	8.31 ± 4.96	6.75 ± 5.38	−1.00 (−4.50, 2.50)	F = 0.33	6.63 ± 4.99	0.93 (−2.04, 3.92)	F = 0.41
TE group	8.19 ± 4.29	7.75 ± 4.26		*p* = 0.565	5.69 ± 3.04		*p* = 0.526
HAQ							
PNE + TE group	9.06 ± 4.65	7.00 ± 4.21	−2.50 (−5.89,0.89)	F = 2.26	6.37 ± 5.45	−2.13 (−5.67, 1.42)	F = 1.49
TE group	9.88 ± 3.57	9.50 ± 5.13		*p* = 0.143	8.50 ± 4.32		*p* = 0.231

ITT: Intention-to-treat; PNE+ET: pain neurophysiology education + exercise therapy; ET: exercise therapy; VAS: visual analogue scale; FIQ-R: Revised Fibromyalgia Impact Questionnaire; PCS: Pain Catastrophizing Scale; HADS: Hospital Anxiety and Depression Scale; HAQ: Health Assessment Questionnaire.

## Supplementary Materials

The following are available online at https://www.mdpi.com/2077-0383/9/11/3564/s1, Supplementary Data: Within-group changes score on ITT analysis.

## Appendix A. Therapeutic Exercise Protocol

**Part 1. Warm-Up**	**Part 2. Central Part**	**Part 3. Cool Down**
10 min: Joint mobility exercises in the available range of motion of: - Hip flexion-extension-abduction-rotations - Knee flexion-extension - Ankle flexion-extension inversion-eversion - Shoulder flexion-extension-abduction-rotations - Lumbar spine flexion-extension-lateral flexion-rotation - Cervical spine flexion-extension-lateral flexion-rotation Dynamic stretching of main muscle groups. Submaximal repetitions of the strengthening exercises.	40 min: **Aerobic exercises** (20 min): Exercises: - Cycling - Walking - Playing games Intensity: Low to moderate intensity (40–60% of maximum heart rate). **Strengthening exercises** (20 min): Circuits of 4 exercises with 3 sets of 10–12 repetitions or 3 sets of 30 s for planks. Exercises: Lower limb exercises: - Squat - Lunge - Glute bridge - Side glute bridge Upper limb exercises: - Biceps brachii curl - Triceps brachii press-down - Rowing with TheraBand - Internal and external rotation with TheraBand - Resistant band side raises Trunk exercises - Push-ups - Planks - Lateral planks Intensity: The intensity was 50% of 1RM. The intensity was increased by 10% according to the individual tolerance. Progression: Adding soft elastic bands and dumbbells.	10 min: Pulmonary exercises: Inspiration through the noise and expiration through the mouth, deep breathing and then expiration through the mouth slowly deep breathing. Stretching exercises: 3 sets of 30 s. Exercises: - Gluteus - Quadriceps femoris - Hamstrings - Biceps brachii - Triceps brachii - Deltoid - Latissimus dorsi
