# Supplementary material for: Therapeutic Exercise and Pain Neurophysiology Education in Female Patients with Fibromyalgia Syndrome: A Feasibility Study
Source: J Clin Med. 2020 Nov 5;9(11):3564. doi: 10.3390/jcm9113564 (PMC7694507; doi:10.3390/jcm9113564)
Supplement: Supplementary file 1 [file jcm-09-03564-s001.pdf]

Supplementary Data. Within-group changes score on ITT analysis.

	Within-group changes score at the end of treatment (T0–T1)				Within-group changes score at 3 months of follow-up (T0–T2)			
	Mean difference	95%CI	*p*-value	Effect size	Mean difference	95%CI	*p*-value	Effect size
VAS last three days (0-10 cm)								
PNE + TE group	2.92	1.73, 4.11	<0.001	1.50	2.70	0.95, 4.44	0.048	1.15
TE group	0.96	−0.35, 2.28	0.205	0.45	1.50	−0.90, 3.10	0.069	0.78
Positive points								
PNE + TE group	2.93	0.36, 5.51	0.023	0.93	3.75	1.33, 6.16	0.002	1.19
TE group	1.81	−0.30, 3.91	0.108	0.67	2.43	0.64, 4.22	0.007	1.00
Algometry (Kg/cm^2)								
PNE + TE group	-19.82	−28.78, -10.85	<0.001	−1.12	-24.36	−35.17, −13.54	<0.001	−1.33
TE group	-12.46	−22.32, -2.60	0.012	−0.82	-13.21	−19.84, -6.57	<0.001	−0.95
FIQ-R								
PNE + TE group	9.1	1.08,19.28	0.077	0.4	23.31	12.93,33.7	<0.001	1.2
TE group	8.02	−1.04,17.09	0.093	0.4	11.68	3.57,19.79	0.008	0.6
PCS Total score								
PNE + TE group	6.06	−1.04, 13.16	0.109	0.50	7.00	1.00, 12.99	0.020	0.57
TE group	3.58	−1.23, 8.35	0.191	0.38	3.06	−1.20, 7.32	0.216	0.34
PCS Rumiation								
PNE + TE group	2.00	−0.28, 4.28	0.096	0.49	2.31	0.36, 4.26	0.018	0.57
TE group	1.56	−0.37, 3.49	0.138	0.43	1.37	−0.51, 3.26	0.700	0.39
PCS Magnification								
PNE + TE group	1.56	−0.14, 3.26	0.078	0.53	1.56	−0.12, 3.24	0.074	0.51
TE group	0.31	−0.41, 1.03	0.793	0.12	0.31	−0.93, 1.56	1	0.13
PCS Helplessness								
PNE + TE group	2.50	−0.80, 5.80	0.180	0.44	3.12	0.20, 6.04	0.034	0.54
TE group	1.75	−0.88, 4.38	0.280	0.38	1.06	−1.06, 3.18	0.592	0.23
HADS total score								
PNE + TE group	3.50	−0.80, 7.80	0.135	0.4	3.87	−0.60, 8.35	0.103	0.48
TE group	2.50	−1.21, 6.21	0.269	0.31	5.62	2.39, 8.85	0.001	0.88
HADS anxiety								
PNE + TE group	1.31	−1.47, 4.10	0.673	0.34	2.18	−0.52, 4.89	0.128	0.60
TE group	1.43	−0.36, 3.24	0.147	0.37	3.12	−0.36, 3.24	0.001	0.95
HADS depression								
PNE + TE group	1.56	−0.40, 3.52	0.148	0.30	1.68	−0.43, 3.81	0.148	0.33
TE group	0.43	−1.28, 2.15	1	0.10	2.50	0.32, 4.67	0.022	0.67
HAQ								

PNE + TE group	2.08	0.04, 4.08	0.045	0.46	2.68	−0.92, 5.46	0.060	0.53
TE group	0.37	−1.44, 2.19	1	0.08	1.37	−0.03, 2.78	0.057	0.34

ITT: Intention-to-treat; PNE+ET: Pain Neurophysiology Education + Exercise Therapy; ET: Exercise Therapy; VAS: Visual Analogue Scale; FIQ-R: Fibromyalgia Impact Questionnaire Revised; PCS: Pain Catastrophizing Scale; HADS: Hospital Anxiety and Depression Scale; HAQ: Health Assessment Questionnaire.
